# Trastuzumab deruxtecan in HER2-positive advanced breast cancer with or without brain metastases: a phase 3b/4 trial

**DOI:** 10.1038/s41591-024-03261-7

**Published:** 2024-09-13

**Authors:** Nadia Harbeck, Eva Ciruelos, Guy Jerusalem, Volkmar Müller, Naoki Niikura, Giuseppe Viale, Rupert Bartsch, Christian Kurzeder, Michaela J. Higgins, Roisin M. Connolly, Sally Baron-Hay, María Gión, Valentina Guarneri, Giampaolo Bianchini, Hans Wildiers, Santiago Escrivá-de-Romaní, Manoj Prahladan, Helen Bridge, Nataliya Kuptsova-Clarkson, Nana Scotto, Sunil Verma, Nancy U. Lin, the DESTINY-Breast12 study group, Nadia Harbeck, Eva Ciruelos, Volkmar Müller, Naoki Niikura, Christian Kurzeder, Michaela J. Higgins, Roisin M. Connolly, Sally Baron-Hay, Valentina Guarneri, Giampaolo Bianchini, Hans Wildiers, Nancy U. Lin, Belinda Yeo, Nicole McCarthy, Amelia McCartney, Timothy Clay, Nicholas Murray, Andrea Gombos, Joëlle Collignon, Eveline De Cuypere, Katarzyna Jerzak, Stephen Chia, Maja Maraldo, Jeanette Rønlev, Eva Brix, Minna Tanner, Johanna Mattson, Riikka Huovinen, Pauline Wimberger, Mattea Reinisch, Hans Tesch, Michael Untch, Peter Fasching, Tjoung-Won Park-Simon, Joke Tio, Michael Braun, Eva Maria Grischke, Frederik Marmé, Marion van Mackelenbergh, John McCaffrey, Michelino De Laurentiis, Michele Caruso, Rossana Berardi, Laura Biganzoli, Vittoria Fotia, Toshinari Yamashita, Junji Tsurutani, Koichiro Tsugawa, Nobumoto Tomioka, Maaike de Boer, Daniel Houtsma, Martin Pilskog, Olav Engebråten, Anna Sætersdal, Piotr Wysocki, Zbigniew Nowecki, Barbara Radecka, Jacek Jassem, Renata Duchnowska, Joana Simões, Fatima Cardoso, Ana Rita Sousa, María Jesús Vidal Losada, Cristina Saura Manich, Joaquin Gavilá Gregori, Maria Pilar Lopez Marti, Manuel Ruiz Borrego, Javier Cortés Castán, Rafael López López, César Rodríguez Sanchez, Encarnación González Flores, Carmen Hinojo González, Purificación Martínez del Prado, Aglaia Schiza, Fredrika Killander, Leif Klint, Lorenzo Rossi, Stefan Aebi, Khalil Zaman, Peter Hall, Carey Anders

**Affiliations:** 1 Breast Center, Department of Gynecology and Obstetrics, Comprehensive Cancer Center Munich, LMU University Hospital, Munich, Germany; 2 Hospital Universitario 12 de Octubre, Madrid, Spain; 3 CHU Liège and Liège University, Liège, Belgium; 4 University Medical Center Hamburg-Eppendorf, Hamburg, Germany; 5 Tokai University School of Medicine, Kanagawa, Japan; 6 Department of Pathology and Laboratory Medicine, IEO European Institute of Oncology IRCCS, Milan, Italy; 7 Division of Oncology, Department of Medicine 1, Medical University of Vienna, Vienna, Austria; 8 Breast Center, University Hospital Basel, Basel, Switzerland; 9 St. Vincent’s University Hospital, UCD Cancer Trials Cluster, Dublin, Ireland; 10 Cancer Research @UCC, College of Medicine and Health, University College Cork, Cork, Ireland; 11 Cancer Trials Cork, CUH/UCC Cancer Center, Cork University Hospital, Cork, Ireland; 12 Department of Medical Oncology, Royal North Shore Hospital, St Leonards, NSW Australia; 13 IOB-Madrid, Beata María Ana Hospital, Madrid, Spain; 14 Department of Medical Oncology, Ramón y Cajal University Hospital, Madrid, Spain; 15 Department of Surgery, Oncology and Gastroenterology, University of Padova, Padova, Italy; 16 Medical Oncology 2, Istituto Oncologico Veneto IRCCS, Padova, Italy; 17 Department of Medical Oncology, IRCCS Ospedale San Raffaele, Milan, Italy; 18 School of Medicine and Surgery, Vita-Salute San Raffaele University, Milan, Italy; 19 Department of General Medical Oncology, University Hospitals Leuven, KU Leuven, Leuven, Belgium; 20 Vall d’Hebron University Hospital, Vall d’Hebron Institute of Oncology, Barcelona, Spain; 21 Global Medical Affairs, Oncology R&D, AstraZeneca, Cambridge, UK; 22 Oncology Global Medical Affairs / Payer Biometrics, AstraZeneca, Macclesfield, UK; 23 Patient Safety, Oncology R&D, AstraZeneca, Gaithersburg, MD USA; 24 Oncology Global Medical Affairs, AstraZeneca, Baar, Switzerland; 25 Oncology Franchise, AstraZeneca, Gaithersburg, MD USA; 26 Department of Medical Oncology, Dana-Farber Cancer Institute, Boston, MA USA; 27 Austin Health, Melbourne, Australia; 28 ICON Cancer Care, Wesley, Australia; 29 Monash Medical Centre, Clayton, Australia; 30 St John of God, Subiaco Hospital, Subiaco, Australia; 31 GenesisCare, Adelaide, Australia; 32 Institut Jules Bordet, Brussels, Belgium; 33 CHU de Liège, Liège, Belgium; 34 AZ Sint-Jan Brugge-Oostende AV, Bruges, Belgium; 35 Sunnybrook Health Sciences Centre, Toronto, Canada; 36 BC Cancer Agency, Vancouver, Canada; 37 Rigshospitalet, Copenhagen, Denmark; 38 Odense University Hospital, Odense, Denmark; 39 Herlev og Gentofte Hospital, Copenhagen, Denmark; 40 TAYS Sydänkeskus Oy, Tampere, Finland; 41 HUS, Helsinki, Finland; 42 Turku University Hospital, Turku, Finland; 43 Universitätsklinikum Carl Gustav Carus der TU Dresden, Dresden, Germany; 44 Kliniken Essen Mitte, Essen, Germany; 45 Centrum für Hämatologie und Onkologie Bethanien, Frankfurt, Germany; 46 Helios-Kliniken Berlin – Buch, Berlin, Germany; 47 Universitätsklinikum Erlangen, Erlangen, Germany; 48 Medizinische Hochschule Hannover, Hannover Medical School, Hannover, Germany; 49 Universitätsklinikum Münster, Münster, Germany; 50 Rotkreuzklinikum München, Munich, Germany; 51 Universitätsklinikum Tübingen, Tübingen, Germany; 52 Medizinische Fakultät Mannheim der Universität Heidelberg, Heidelberg, Germany; 53 Universitätsklinikum Schleswig-Holstein Campus Kiel, Kiel, Germany; 54 Mater Misericordiae University Hospital, Dublin, Ireland; 55 Istituto Nazionale Tumori Fondazione Pascale IRCCS, Naples, Italy; 56 Humanitas Istituto Clinico Catanese, Catania, Italy; 57 AOU Ospedali Riuniti Umberto I – G.M. Lancisi – G. Salesi, Ancona, Italy; 58 Nuovo Ospedale di Prato, Prato, Italy; 59 A.O. Papa Giovanni XXIII, Bergamo, Italy; 60 Kanagawa Cancer Center, Kanagawa, Japan; 61 Showa University Hospital, Shinagawa, Japan; 62 St. Marianna University Hospital, Kawasaki, Japan; 63 National Hospital Organization Hokkaido Cancer Center, Sapporo, Hokkaido, Japan; 64 Maastricht University Medical Center, Maastricht, Netherlands; 65 HAGA Ziekenhuis locatie Els Borst-Eilersplein, Hague, Netherlands; 66 Helse Bergen HF Haukeland universitetssykehus, Bergen, Norway; 67 Oslo University Hospital, Oslo, Norway; 68 Szpital Uniwersytecki w Krakowie, Krakow, Poland; 69 Narodowy Instytut Onkologii im. Marii Skłodowskiej-Curie, Warsaw, Poland; 70 Opolskie Centrum Onkologii im. prof. T. Koszarowskiego, Opole, Poland; 71 Uniwersyteckie Centrum Kliniczne, Gdansk, Poland; 72 Wojskowy Instytut Medyczny, Warsaw, Poland; 73 Centro Hospitalar Universitario de Porto, Porto, Portugal; 74 Fundação Champalimaud, Lisbon, Portugal; 75 Centro Hospitalar Universitario Lisboa Norte, Lisbon, Portugal; 76 Hospital Clínic Barcelona, Barcelona, Spain; 77 Hospital Universitario Vall d’Hebron, Barcelona, Spain; 78 Fundación Instituto Valenciano de Oncología (IVO), Valencia, Spain; 79 Hospital Universitario de la Princesa, Madrid, Spain; 80 Hospital Universitario Virgen del Rocio, Seville, Spain; 81 Hospital Ruber Internacional, Madrid, Spain; 82 Complejo Hospitalario Universitario de Santiago (CHUS), Santiago, Spain; 83 Hospital Clínico Universitario de Salamanca, Salamanca, Spain; 84 Hospital Universitario Virgen de las Nieves, Granada, Spain; 85 Hospital Universitario Marques de Valdecilla, Santander, Spain; 86 Hospital Civil de Basurto, Bilbao, Spain; 87 Akademiska Sjukhuset Uppsala, Uppsala, Sweden; 88 Skånes Universitetssjukhus, Lund, Sweden; 89 Sahlgrenska Universitetssjukhuset, Göteborg, Sweden; 90 Ente Ospedaliero Cantonale, Bellinzona, Switzerland; 91 Luzerner Kantonsspital (LUKS) - Luzern, Luzern, Switzerland; 92 Centre Hospitalier Universitaire Vaudois, Lausanne, Switzerland; 93 Western General Hospital, Edinburgh, United Kingdom; 94 Duke University Medical Center, Durham, NC USA

**Keywords:** Breast cancer, Breast cancer

## Abstract

Trastuzumab deruxtecan (T-DXd) intracranial activity has been observed in small or retrospective patient cohorts with human epidermal growth factor receptor 2–positive (HER2^+^) advanced/metastatic breast cancer (mBC) and stable or active (untreated/previously treated and progressing) brain metastases (BMs). The phase 3b/4 DESTINY-Breast12 study investigated T-DXd in patients with HER2^+^ mBC and is, to our knowledge, the largest prospective study of T-DXd in patients with BMs in this setting. Patients (stable/active BMs (*n* = 263) and no BMs (*n* = 241)) treated with one or more prior anti-HER2–based regimens received T-DXd (5.4 mg per kg). Primary endpoints were progression-free survival (PFS; BMs cohort) and objective response rate (ORR) per Response Evaluation Criteria in Solid Tumors version 1.1 (non-BMs cohort). Additional endpoints included central nervous system (CNS) PFS, ORR, time to second progression, CNS ORR (BMs cohort), incidence of new symptomatic CNS metastases (non-BMs cohort), time to progression, duration of response, overall survival and safety (both cohorts). No formal hypothesis testing was conducted for this single-arm, open-label study. In the BMs cohort, 12-month PFS was 61.6% (95% confidence interval (CI): 54.9–67.6), and 12-month CNS PFS was 58.9% (95% CI: 51.9–65.3). In the non-BMs cohort, ORR was 62.7% (95% CI: 56.5–68.8). Grade 3 or higher adverse events occurred in 51% (BMs cohort) and 49% (non-BMs cohort) of patients. Investigator-reported interstitial lung disease/pneumonitis occurred in 16% (grade ≥3: 3%) of patients with BMs and 13% (grade ≥3: 1%) of patients without BMs. These data show substantial and durable overall and intracranial activity for T-DXd, supporting its use in previously treated patients with HER2^+^ mBC irrespective of stable/active baseline BMs. ClinicalTrials.gov identifier: NCT04739761.

## Abstract (web summary)

In the non-randomized phase 3b/4 DESTINY-Breast12 study, trastuzumab deruxtecan (T-DXd) treatment of patients with HER2^+^ advanced breast cancer and active or stable brain metastases showed consistent intracranial activity and systemic efficacy of T-DXd.

## Main

Human epidermal growth factor receptor 2–positive (HER2^+^) breast cancer accounts for between 15% and 20% of all cases of breast cancer^1,2^. As many as 50% of patients with HER2^+^ advanced/metastatic breast cancer (mBC) develop brain metastases (BMs), which are associated with a poorer prognosis compared to patients who do not have BMs^3–6^.

Local therapy (including surgical resection, stereotactic radiosurgery (SRS), stereotactic radiotherapy and/or whole-brain radiation therapy (WBRT)) is recommended for BMs^7^; however, central nervous system (CNS) progression typically occurs within 6–12 months of treatment, and no extracranial benefit is conferred^8–11^. WBRT, currently recommended for treatment of multiple BMs, is associated with cognitive deterioration^7,12^; as some patients with HER2^+^ breast cancer and BMs can survive for several years, this is of particular concern^13^. As such, additional systemic treatment options for patients with BMs are needed. Trastuzumab-based therapy has long been the mainstay of systemic therapy for patients with HER2^+^ mBC, and several additional HER2-directed therapies have been investigated for the treatment of HER2^+^ mBC with BMs, including tucatinib^14–17^. Despite this, a large proportion of patients receiving treatment, including those with an initial response, eventually experience disease progression (commonly in the CNS)^17^.

Trastuzumab deruxtecan (T-DXd) is an antibody–drug conjugate (ADC) composed of a humanized immunoglobulin G1 monoclonal antibody specifically targeting HER2, a tetrapeptide-based cleavable linker and a potent topoisomerase I inhibitor payload^18,19^. On the basis of results from the randomized phase 3 DESTINY-Breast03 study, T-DXd is approved for the treatment of adult patients with unresectable or metastatic HER2^+^ breast cancer who have received a prior anti-HER2–based regimen in the metastatic setting or who have received a prior anti-HER2–based regimen in the neoadjuvant or adjuvant setting and developed disease recurrence during or within 6 months of completing therapy^20,21^.

Promising preliminary evidence of T-DXd intracranial efficacy was reported in a retrospective, exploratory pooled analysis of DESTINY-Breast01, 02 and 03. Patients with HER2^+^ mBC and stable (*n* = 104) or active (*n* = 44) BMs were treated with T-DXd^22^. The intracranial objective response rate (ORR) was 45.2% in patients with stable BMs and 45.5% in patients with active BMs. Median (95% confidence interval (CI)) CNS progression-free survival (CNS PFS) was 12.3 months (11.1–13.8) in patients with stable BMs and 18.5 months (13.6–23.3) in patients with active BMs^22^. Encouraging intracranial responses in patients with active (untreated or previously treated and progressing) BMs were also reported in the phase 1b/2 DESTINY-Breast07 study (*n* = 35); in the ongoing, five-cohort phase 2 DEBBRAH study (*n* = 13); in ROSET‑BM, a multicenter, retrospective, medical chart review study (*n* = 67); in the prospective, single-arm, single-center, phase 2 TUXEDO‑1 study (*n* = 15); and in a retrospective cohort analysis of heavily pretreated patients with BMs (*n* = 10)^23–27^.

Here we report results from the phase 3b/4 DESTINY-Breast12 study (NCT04739761), a non-comparative study that evaluated the efficacy and safety of T-DXd in patients with HER2^+^ mBC from two separate cohorts of patients with and without baseline BMs. DESTINY-Breast12 is, to our knowledge, the largest prospective study of T-DXd in patients with HER2^+^ mBC with previously treated and stable or active (untreated or previously treated and progressing) BMs.

## Results

### Patients

**Fig. 1:**
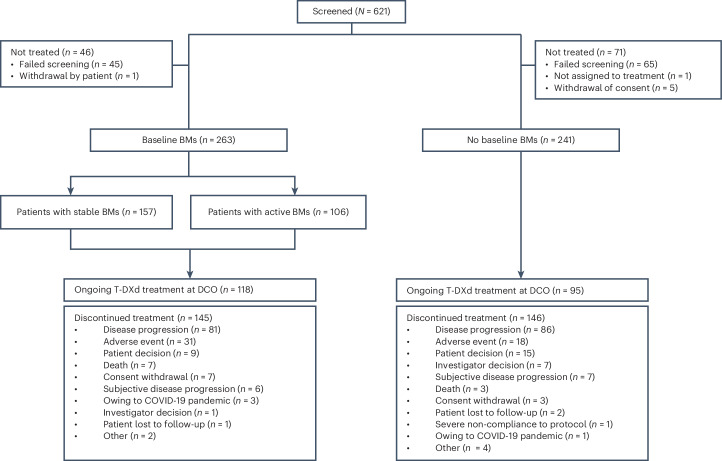
Patient disposition. COVID-19, coronavirus disease 2019; DCO, data cutoff.

A total of 504 patients were treated across 78 sites between June 2021 and February 2024; 263 patients had baseline BMs, and 241 patients had no baseline BMs (Fig. 1). Of patients with baseline BMs, 157 had previously treated and stable BMs, and 106 had active BMs (39 had untreated BMs; 67 had previously treated BMs that were progressive at study entry (hereafter termed previously treated/progressing BMs), with no clinical indication for immediate retreatment with local therapy). Demographics and baseline disease characteristics for both cohorts are summarized in Table 1. Patients with baseline BMs received a median of 1.0 regimen (range, 0–4) of previous anti-cancer therapy in the metastatic setting, and 158 patients (60.1%) received prior CNS radiotherapy (including 40 patients (15.2%) who had WBRT and 15 (5.7%) who had SRS). Median time from last CNS radiotherapy to treatment initiation in patients with prior CNS radiotherapy was 164 d (range, 9–2,115) overall and 116.5 d (range, 9–1,798) and 214.5 d (range, 15–2,115) in the stable (*n* = 90) and active (*n* = 68) BMs subgroups, respectively. The median follow-up duration in this cohort was 15.4 months (range, 0.1–30.0), and 118 patients (44.9%) were continuing to receive treatment at final data cutoff (8 February 2024). The most common reasons for discontinuation of study treatment included progressive disease (PD; 30.8%) and adverse events (AEs; 11.8%) (Fig. 1). Patients with no baseline BMs received a median of 1.0 regimen (range, 0–4) of previous anti-cancer therapy in the metastatic setting and had a median follow-up duration of 16.1 months (range, 0.8–28.4); 95 patients (39.4%) were continuing to receive treatment at final data cutoff. Primary reasons for discontinuation of study treatment included PD (35.7%) and AEs (7.5%) (Fig. 1).

**Table 1:** Demographics and baseline clinical characteristics for patients with and without baseline BMs

	Patients with baseline BMs (*N* = 263)	Patients with no baseline BMs (*N* = 241)
Age, median (range), years	52 (28–86)	54 (24–87)
Female, *n* (%)	263 (100)	241 (100)
Race, *n* (%)		
White	231 (87.8)	209 (86.7)
Black	4 (1.5)	2 (0.8)
Asian	19 (7.2)	15 (6.2)
Other	6 (2.3)	13 (5.4)
Not reported	1 (0.4)	1 (0.4)
Missing	2 (0.8)	1 (0.4)
HER2 status, *n* (%)		
2^+^	2 (0.8)	5 (2.1)
3^+^	187 (71.1)	141 (58.5)
Positive^a^	74 (28.1)	95 (39.4)
HR status, *n* (%)^b^		
HR^+^	165 (62.7)	150 (62.2)
ECOG PS at baseline, *n* (%)		
0	163 (62.0)	194 (80.5)
1	100 (38.0)	47 (19.5)
Metastatic sites at baseline, *n* (%)^c^		
Bone and locomotor	97 (36.9)	85 (35.3)
Liver metastases	58 (22.1)	66 (27.4)
Lung	67 (25.5)	67 (27.8)
Measurable disease at baseline^d^		
Yes	198 (75.3)	215 (89.2)
Prior regimens of anti-cancer therapies for metastatic disease		
Median number of regimens (range)	1.0 (0–4)	1.0 (0–4)
Number of regimens, *n* (%)		
0	20 (7.6)	18 (7.5)
1	132 (50.2)	124 (51.5)
2	109 (41.4)	96 (39.8)
≥3	2 (0.8)	3 (1.2)
Prior HER2 inhibitor agents, *n* (%)^e^	262 (99.6)	240 (99.6)
Trastuzumab	258 (98.1)	233 (96.7)
Pertuzumab	228 (86.7)	207 (85.9)
T-DM1	106 (40.3)	94 (39.0)
Lapatinib	11 (4.2)	13 (5.4)
Neratinib	4 (1.5)	2 (0.8)
Tucatinib^f^	2 (0.8)	0
T-DXd	1 (0.4)	0
Specific agent not reported	1 (0.4)	0
Prior CNS therapies, *n* (%)	168 (63.9)	–
Brain surgery	48 (18.3)	–
CNS radiotheraphy^g^	158 (60.1)	–
WBRT	40 (15.2)	–
SRS	15 (5.7)	–
IMRT	16 (6.1)	–
SBRT	11 (4.2)	–
Stereotactic intracranial radiotherapy	38 (14.4)	–
3D conformal	13 (4.9)	–
Other	44 (16.7)	–
Missing	10 (3.8)	–

ER, estrogen receptor; HR, hormone receptor; IMRT, intensity-modulated radiation therapy; PR, progesterone receptor; SBRT, stereotactic body radiation therapy.

^a^HER2 status is positive if the specific HER2 status is unknown.

^b^HR status is positive if either or both of ER/PR status is positive or is ≥1%. HR status is negative if both ER and PR status are either negative or have a result <1%.

^c^Patients with metastasis in more than one site are counted once in each of those sites.

^d^109/157 patients in the stable BMs subgroup and 89/106 patients in the active BMs subgroup had measurable disease at baseline.

^e^Where two or more treatments had the same month and year for start date but day was missing, the treatments were assumed to be a combination regimen; treatments were not included if only the year was reported (day and month both missing); patients with multiple occurrences for the same HER2 inhibitor or anti-HER2 combination regimen are counted only once for that inhibitor or regimen; patients with occurrences for more than one HER2 inhibitor or anti-HER2 combination regimen are counted once for each of those inhibitors or regimens.

^f^Two patients with prior tucatinib use were recorded as protocol deviations.

^g^151 patients received intracranial radiotherapy and seven were treated with radiotherapy to spinal cord locations only.

Response and progression in both cohorts were assessed by independent central review (ICR) per Response Evaluation Criteria in Solid Tumors version 1.1 (RECIST 1.1).

### Overall efficacy in the baseline BMs cohort

**Fig. 2:**
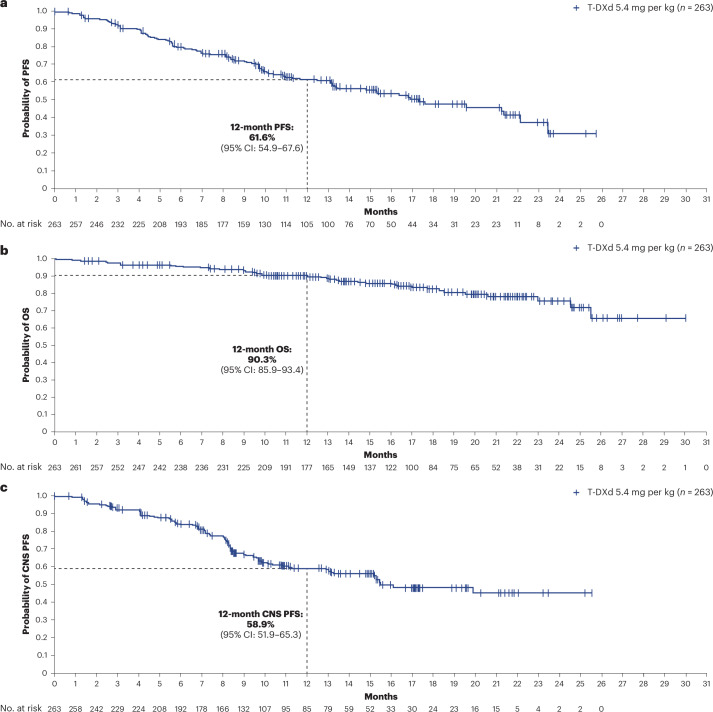
Kaplan–Meier analysis of key efficacy endpoints in patients with baseline BMs. **a**, Overall PFS. **b**, OS. **c**, CNS PFS per RECIST 1.1 as assessed by ICR. Tick marks indicate censored data. Analysis was based on the full analysis set.

**Fig. 3:**
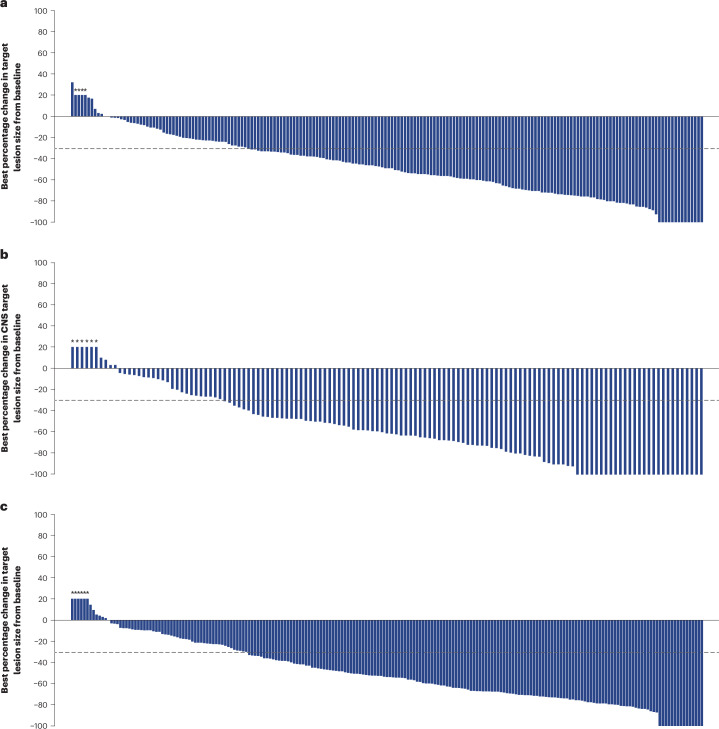
Best percentage change in target lesions. **a**, Best percentage change from baseline in target lesion size in patients with baseline BMs and measurable disease at baseline (full analysis set). **b**, Best percentage change from baseline in CNS target lesion size in patients with baseline BMs and measurable CNS disease at baseline. **c**, Best percentage change from baseline in target lesion size, in patients with no baseline BMs and measurable disease at baseline (full analysis set). All patients had at least one post-baseline scan. Responses were assessed per RECIST 1.1 by ICR. A value of +20% was imputed as best percentage change from baseline if best percentage change could not be calculated because of missing data in the following situations: a patient had a new lesion or progression of non-target lesions or target lesions or a patient had withdrawn because of PD and had no evaluable target lesion data before or at PD. The dashed line indicates a 30% decrease in tumor size (partial response). Asterisks indicate imputed values.

**Extended Data Fig. 1:**
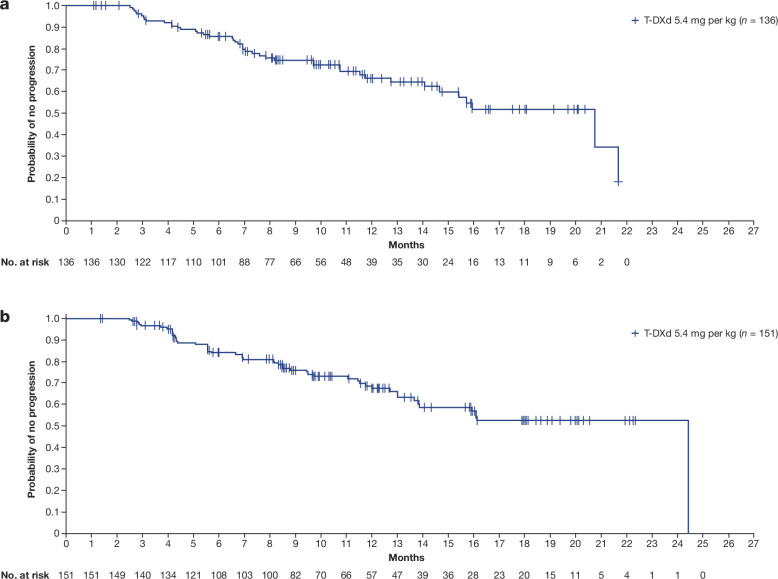
Kaplan-Meier analysis of duration of response. Duration of response per RECIST 1.1 by ICR in all patients with a confirmed complete or partial response. **a**, Patients with baseline BMs. **b**, Patients with no baseline BMs. Tick marks indicate censored data. BM, brain metastasis; ICR, independent central review; RECIST 1.1, Response Evaluation Criteria in Solid Tumors version 1.1.

Overall PFS at 12 months was 61.6% (95% CI: 54.9–67.6) in all patients with BMs (Fig. 2a and Table 2) and 62.9% (95% CI: 54.0–70.5) and 59.6% (95% CI: 49.0–68.7) in patients with stable and active BMs, respectively (Table 2). Within the active BMs subgroup, PFS at 12 months was 47.0% (95% CI: 29.6–62.7) and 66.7% (95% CI: 53.4–76.9) in patients with untreated and previously treated/progressing BMs, respectively (post hoc analysis). Overall, 89 patients (33.8%) were free of progression at the time of the analysis, and median PFS (post hoc analysis) was 17.3 months (95% CI: 13.7–22.1). Time to progression (time from first dose until documented disease progression) data were immature, and the median was not calculated. Time to second progression (PFS2; time from first dose to second progression or death) data were immature, and the median was not reached. PFS2 at 12 months was 83.1% (95% CI: 77.5–87.4). Overall survival (OS) data were immature at the time of analysis (16.3% maturity); 12-month OS was 90.3% (95% CI: 85.9–93.4) (Fig. 2b and Table 2). In the baseline BMs full analysis set, confirmed ORR was 51.7% (95% CI: 45.7–57.8) (Table 2). A total of 11 patients (4.2%) had a complete response, and 125 (47.5%) patients had a partial response. Most responses (121/136) were reported by 6 months; at the time of analysis, response was ongoing in more than 50% of patients (*n* = 134 (51.0%)), and, therefore, median duration of response (DOR) was not calculated (Extended Data Fig. 1a). The ORR in patients with stable and active BMs was 49.7% (95% CI: 41.9–57.5) and 54.7% (95% CI: 45.2–64.2), respectively (Table 2). In a post hoc analysis restricted to patients with measurable disease at baseline (*n* = 198), confirmed ORR was 64.1% (95% CI: 57.5–70.8) overall and 67.0% (95% CI: 58.1–75.8) and 60.7% (95% CI: 50.5–70.8) in patients with stable (*n* = 109) and active (*n* = 89) BMs, respectively (Table 2). The best percentage change in target lesion size is shown in Fig. 3a.

**Table 2:** Overall anti-tumor activity in patients with and without baseline BMs

	Patients with baseline BMs (*N* = 263)			Patients with no baseline BMs (*N* = 241)
	Overall (*N* = 263)	Stable BMs (*n* = 157)	Active BMs (*n* = 106)	Overall (*N* = 241)
12-month PFS, % (95% CI)^a^	61.6 (54.9–67.6)	62.9 (54.0–70.5)	59.6 (49.0–68.7)	–
12-month CNS PFS, % (95% CI)^a^	58.9 (51.9–65.3)	57.8 (48.2–66.1)	60.1 (49.2–69.4)	–
Median PFS, months (95% CI)^b^	17.3 (13.7–22.1)	NR	NR	–
12-month OS, % (95% CI)	90.3 (85.9–93.4)	93.2 (87.7–96.3)	86.1 (77.6–91.5)	90.6 (86.0–93.8)
Confirmed ORR, % (95% CI)^a,c^	51.7 (45.7–57.8)	49.7 (41.9–57.5)	54.7 (45.2–64.2)	62.7 (56.5–68.8)
Best objective response, *n* (%)^a,c^				
Complete response	11 (4.2)	NR	NR	23 (9.5)
Partial response	125 (47.5)	NR	NR	128 (53.1)^d^
Stable disease ≥5 weeks	110 (41.8)	NR	NR	76 (31.5)
Progressive disease	9 (3.4)	NR	NR	8 (3.3)
Not evaluable	8 (3.0)	NR	NR	6 (2.5)
Confirmed ORR in patients with measurable disease only, % (95% CI)^a,b,c,e^	64.1 (57.5–70.8)	67.0 (58.1–75.8)	60.7 (50.5–70.8)	68.4 (62.2–74.6)
Best objective response in patients with measurable disease, *n* (%)^a,b,c,e^				
Complete response	2 (1.0)	NR	NR	20 (9.3)
Partial response	125 (63.1)	NR	NR	127 (59.1)
Stable disease ≥5 weeks	61 (30.8)	NR	NR	57 (26.5)
Progressive disease	4 (2.0)	NR	NR	6 (2.8)
Not evaluable	6 (3.0)	NR	NR	5 (2.3)
Confirmed CNS ORR, % (95% CI)^a,c,f^	71.7 (64.2–79.3)	79.2 (70.2–88.3)	62.3 (50.1–74.5)	–
Incidence of new symptomatic CNS metastasis, *n*, incidence rate (95% CI)	–	–	–	4, 0.017 (0.00452–0.04250)

NR, not reported.

Analysis was based on the full analysis set. Response and disease progression were assessed by RECIST 1.1.

^a^By ICR.

^b^Post hoc analysis.

^c^Response requires confirmation by repeat imaging no less than 4 weeks after the visit when response was first observed.

^d^One patient with no measurable disease at baseline was assigned partial response by ICR.

^e^Patients with measurable disease at baseline: *n* = 198 (baseline BMs); *n* = 109 (stable BMs); *n* = 89 (active BMs); *n* = 215 (no baseline BMs).

^f^Patients with measurable CNS disease at baseline: *n* = 138 (baseline BMs); *n* = 77 (stable BMs); *n* = 61 (active BMs).

### CNS efficacy in the baseline BMs cohort

Overall, CNS (including intracranial) progression was reported in 101 (38.4%) patients treated with T-DXd; CNS PFS at 12 months was 58.9% (95% CI: 51.9–65.3) (Fig. 2c and Table 2) and 57.8% (95% CI: 48.2–66.1) and 60.1% (95% CI: 49.2–69.4) in patients with stable and active BMs, respectively (Table 2). In total, 138 (52.5%) patients had measurable CNS disease at baseline (stable BMs: *n* = 77; active BMs: *n* = 61). The proportion of these patients with confirmed CNS ORR overall was 71.7% (95% CI: 64.2–79.3) and 79.2% (95% CI: 70.2–88.3) and 62.3% (95% CI: 50.1–74.5) in patients with stable and active BMs, respectively (Table 2). Within the active BMs subgroup, CNS ORR was reported in 19 out of 23 patients (82.6% (95% CI: 67.1–98.1)) and in 19 out of 38 patients (50.0% (95% CI, 34.1–65.9)) with untreated and previously treated/progressing BMs, respectively (post hoc analysis). The best percentage change in CNS target lesion size is shown in Fig. 3b.

### Overall efficacy in the no baseline BMs cohort

**Extended Data Fig. 2:**
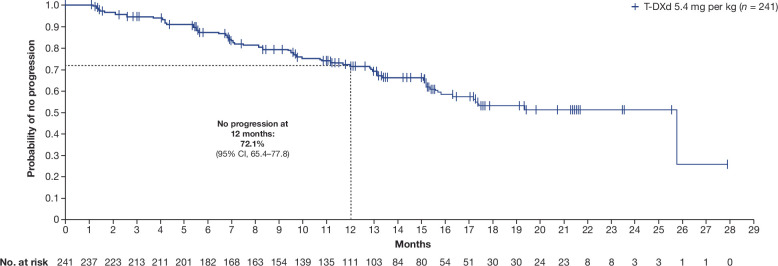
Kaplan-Meier analysis of time to progression in patients with no baseline BMs. Time to progression per RECIST 1.1 as assessed by ICR in patients from the full analysis set. Tick marks indicate censored data. BM, brain metastasis; ICR, independent central review; RECIST 1.1, Response Evaluation Criteria in Solid Tumors version 1.1.

The proportion of patients in the no baseline BMs full analysis set with confirmed ORR was 62.7% (95% CI: 56.5–68.8) (Table 2). A total of 23 patients (9.5%) had a complete response, and 128 (53.1%) patients had a partial response (Table 2). Most responses (140/151) were reported by 6 months; at the time of analysis, response was ongoing in more than 50% of patients (*n* = 151 (62.7%)), and, therefore, median DOR was not calculated (Extended Data Fig. 1b). In a post hoc analysis of patients with measurable disease at baseline (*n* = 215), confirmed ORR was 68.4% (95% CI: 62.2–74.6) (Table 2). The best percentage change in target lesion size is shown in Fig. 3c. OS data were immature at the time of analysis (17.0% maturity); 12-month OS was 90.6% (95% CI: 86.0–93.8) (Table 2). At 12 months, 72.1% (95% CI: 65.4–77.8) of patients had not experienced progression; time to progression data were immature, and the median was not calculated (Extended Data Fig. 2). Only four patients developed new symptomatic CNS metastases (incidence rate 0.017%; 95% CI: 0.00452–0.04250) (Table 2).

### Safety: baseline BMs cohort

Median total treatment duration was 11.5 months (range, 0.1–26.9; Extended Data Table 1). The most common AEs included nausea, fatigue and constipation (Table 3). Grade 3 or higher AEs occurred in 134 (51.0%) patients, and the most common grade 3 or higher AEs were neutropenia (*n* = 43 (16.3%)), fatigue (*n* = 23 (8.7%)) and anemia (*n* = 19 (7.2%)). AEs led to treatment discontinuation in 40 (15.2%) patients (Extended Data Table 1); the most common AE leading to discontinuation was interstitial lung disease (ILD)/pneumonitis (*n* = 27 (10.3%)). Investigator-reported ILD/pneumonitis occurred in 42 patients (16.0%) with baseline BMs; most events were grade 1 (*n* = 26 (9.9%)), and there were six (2.3%) grade 5 events (Extended Data Tables 2 and 3). The median time to first onset of ILD/pneumonitis was 168.0 d (range, 35–646). Seven patients (2.7%) had a reported opportunistic infection (no systematic testing for infection was done); five patients had opportunistic infection reported as co-occurring with ILD/pneumonitis (ILD/pneumonitis events were grade 4 (*n* = 1) or grade 5 (*n* = 4); opportunistic infections were aspergillus (*n* = 1) and *Pneumocystis jirovecii* pneumonia (PJP)/infection (*n* = 4)). Two patients had opportunistic infections that were not reported to co-occur with ILD/pneumonitis (cytomegalovirus infection (*n* = 1) and PJP (*n* = 1)). Left ventricular ejection fraction decrease from baseline occurred in 31 patients (11.8%); no grade 4 or higher events were reported (Extended Data Table 2).

**Table 3:** Most common AEs by grouped and preferred term (≥20% of patients in either cohort)

	Patients with baseline BMs (*N* = 263)		Patients with no baseline BMs (*N* = 241)	
	All grades, *n* (%)	Grade ≥3, *n* (%)	All grades, *n* (%)	Grade ≥3, *n* (%)
Any AE	259 (98.5)	134 (51.0)	237 (98.3)	118 (49.0)
Nausea	172 (65.4)	12 (4.6)	171 (71.0)	12 (5.0)
Fatigue^a^	164 (62.4)	23 (8.7)	176 (73.0)	24 (10.0)
Constipation	105 (39.9)	1 (0.4)	98 (40.7)	1 (0.4)
Neutropenia^b^	80 (30.4)	43 (16.3)	87 (36.1)	44 (18.3)
Alopecia^c^	75 (28.5)	4 (1.5)	83 (34.4)	0
Diarrhea	74 (28.1)	4 (1.5)	86 (35.7)	7 (2.9)
Musculoskeletal pain^d^	73 (27.8)	1 (0.4)	63 (26.1)	2 (0.8)
Vomiting	73 (27.8)	3 (1.1)	81 (33.6)	7 (2.9)
Anemia^e^	70 (26.6)	19 (7.2)	63 (26.1)	12 (5.0)
Upper respiratory tract infection^f^	67 (25.5)	1 (0.4)	50 (20.7)	1 (0.4)
Transaminases increased^g^	60 (22.8)	11 (4.2)	53 (22.0)	8 (3.3)
Headache^h^	57 (21.7)	1 (0.4)	49 (20.3)	4 (1.7)
Decreased appetite	54 (20.5)	4 (1.5)	44 (18.3)	0

Analysis was based on the safety analysis set. Number (%) of patients with AEs, sorted in decreasing frequency for grouped/preferred term in the baseline BMs cohort. Each patient is represented with the maximum reported CTCAE 5.0 grade for each grouped/preferred term. Patients with events in more than one grouped/preferred term are counted once in each of those grouped/preferred terms. Includes AEs with an onset date or worsening on or after the date of first dose and up to 47 d after last dose of study treatment or before the start of new anti-cancer therapies, whichever occurred earlier.

^a^Includes the preferred terms asthenia, fatigue, lethargy and malaise.

^b^Includes the preferred terms neutropenia and neutrophil count decreased.

^c^Grade 3 alopecia is an investigator input error.

^d^Includes the preferred terms back pain, bone pain, limb discomfort, muscle spasms, musculoskeletal chest pain, musculoskeletal pain, myalgia, neck pain and pain in extremity.

^e^Includes the preferred terms anemia, hemoglobin decreased and red blood cell count decreased.

^f^Includes the preferred terms influenza, influenza-like illness, laryngitis, nasopharyngitis, pharyngitis, rhinitis, sinusitis and upper respiratory tract infection.

^g^Includes the preferred terms alanine aminotransferase increased, aspartate aminotransferase increased, gamma-glutamyltransferase increased, hypertransaminasemia, liver function test abnormal and transaminases increased.

^h^Includes the preferred terms headache, migraine and sinus headache.

### Safety: no baseline BMs cohort

Median total treatment duration was 12.0 months (range, 0.7–28.4; Extended Data Table 1). The most common AEs included fatigue, nausea and constipation (Table 3). Grade 3 or higher AEs occurred in 118 (49.0%) patients, and the most common grade 3 or higher AEs were neutropenia (*n* = 44 (18.3%)), fatigue (*n* = 24 (10.0%)) and anemia (*n* = 12 (5.0%)). AEs led to treatment discontinuation in 23 (9.5%) patients (Extended Data Table 1); the most common AE leading to discontinuation was ILD/pneumonitis (*n* = 13 (5.4%)). Investigator-reported ILD/pneumonitis occurred in 31 (12.9%) patients with no baseline BMs; most events were grade 1 (*n* = 22 (9.1%)), and there were three (1.2%) grade 5 events (Extended Data Tables 2 and 3). The median time to first onset of ILD/pneumonitis was 169.0 d (range, 24–484). Left ventricular ejection fraction decrease occurred in 26 (10.8%) patients; there were no grade 3 or higher events (Extended Data Table 2).

## Discussion

DESTINY-Breast12 is, to our knowledge, the largest prospective study reporting intracranial activity of T-DXd in patients with HER2^+^ mBC and baseline BMs. This phase 3b/4 study was designed to collect data from settings that resemble real-world clinical practice, to provide a detailed understanding of T-DXd outcomes in patients previously treated with HER2-targeted agents.

PFS was selected as the primary endpoint in the BMs cohort because it was anticipated that a large proportion of this patient population may have no measurable disease at baseline and to minimize any potential confounding effect from prior locally directed therapy^28^. The 12-month overall PFS rate was 61.6% (95% CI: 54.9–67.6) in patients with baseline BMs. Overall ORR (including patients with no measurable disease at baseline) was lower in patients with stable BMs (49.7%) compared to patients with active BMs (54.7%); however, in line with clinical expectations, a post hoc analysis of ORR in patients with measurable disease at baseline revealed a higher ORR in the stable BMs subgroup (67.0%) versus the active BMs subgroup (60.7%). The different trends observed are likely explained by the imbalance of patients with measurable disease between the two subgroups.

Currently, tucatinib in combination with trastuzumab and capecitabine is the preferred systemic therapy for previously treated patients with HER2^+^ mBC and active BMs^7,29,30^. The phase 2 HER2CLIMB study investigated trastuzumab and capecitabine with either placebo or tucatinib in patients with previously treated HER2^+^ mBC. Patients in the tucatinib arm of the total population (*N* = 612) were heavily pretreated at baseline (median of three (range, 1–14) previous therapy regimens in the metastatic setting^31,32^). In an updated exploratory subanalysis of patients with measurable baseline BMs (*n* = 198), confirmed intracranial ORR was 47.3% (95% CI: 33.7–61.2) in patients with active BMs receiving tucatinib, capecitabine and trastuzumab. Median CNS PFS by investigator per RECIST 1.1 was 9.9 months (95% CI: 8.4–11.7) overall and 9.6 months (95% CI: 7.6–11.1) and 13.9 months (95% CI: 9.7–24.9) in patients with active and stable BMs, respectively^32^. In DESTINY-Breast12, tucatinib as a previous regimen was exclusionary, to avoid any confounding effect from a drug known to be active on CNS lesions.

Because of the decreased quality of life and poor prognosis observed in patients with BMs^3–6^, additional treatment options for this patient population are needed, particularly for later lines of therapy. CNS activity with tucatinib (a small-molecule tyrosine kinase inhibitor) is well established; however, questions remain regarding the intracranial efficacy of ADCs, including T-DXd, in patients with active BMs. Despite its large molecular size, promising CNS activity for T-DXd was previously reported. The 12-month PFS for the overall BMs population in DESTINY-Breast12 was similar to that observed in an exploratory analysis of patients with stable and active BMs enrolled in DESTINY-Breast03 (72.0% (95% CI: 55.0–83.5); *n* = 43), a phase 3, randomized, open-label study that investigated T-DXd versus trastuzumab emtansine (T-DM1) in patients with HER2^+^ mBC previously treated with trastuzumab and a taxane^33^.

Promising CNS activity in patients with active BMs treated with T-DXd was observed in small prospective studies^24,26^. In the phase 2 DEBBRAH (*n* = 13) and TUXEDO-1 (*n* = 15) studies, intracranial ORR per Response Assessment in Neuro-Oncology (RANO)-BM criteria was 46.2% (95% CI: 19.2–74.9) and 73.3% (95% CI: 48.1–89.1), respectively^24,26^. In an interim analysis of the dose-expansion phase of the ongoing DESTINY-Breast07 study (*n* = 35), which assessed T-DXd monotherapy in patients with HER2^+^ mBC and active BMs in the first-line or second-line setting, PFS at 12 months was 75.0% (80% CI: 63.5–83.4), and median PFS was 19.5 months (80% CI: 19.4–24.3) (Anders et al.^23^). Results from DESTINY-Breast12 extend these observations to a larger group of patients with active BMs (12-month PFS: 59.6% (95% CI: 49.0–68.7)), including those with untreated BMs (47.0% (95% CI, 29.6–62.7)) and previously treated/progressing BMs (66.7% (95% CI, 53.4–76.9)).

CNS ORR in our study was 71.7% overall and 79.2% and 62.3% in patients with stable and active BMs, respectively. Within the active BMs subgroup, CNS ORR was 82.6% in patients with untreated BMs and 50.0% in patients with previously treated/progressing BMs. These results are numerically higher than those observed in the pooled DESTINY-Breast01, 02 and 03 analysis of patients with treated/stable (45.2%; *n* = 104) or active (45.5%; *n* = 44) BMs^22^. This may be reflective of the heavily pretreated population included in the pooled analysis (median 3.0 prior treatment regimens in the metastatic setting versus 1.0 for DESTINY-Breast12). In DESTINY-Breast12, responses in patients with baseline BMs were durable, despite a relatively short follow-up duration (15.4 months).

Results from clinical studies have also been corroborated by real-world evidence. In the retrospective ROSET-BM study, 12-month PFS was 62.0% (95% CI: 47.8–73.4) in patients with active BMs (*n* = 67) and 71.4% (95% CI: 33.7–90.1) in patients with stable BMs (*n* = 12), in line with the results of the current study^25^. Patients with leptomeningeal metastases (LM) were excluded from DESTINY-Breast12. However, T-DXd showed sustained activity in two small retrospective studies in patients with mBC and LM: ROSET-BM (*n* = 19) and a small case series (*n* = 8)^25,34^. Further investigation is needed to confirm the efficacy of T-DXd in this patient population.

ORR was chosen as the primary endpoint for the cohort of patients without baseline BMs as it is an early indicator of treatment effect and allowed for early assessment of T-DXd benefit. In this cohort, overall efficacy was in line with prior reports; however, the proportion of patients with complete responses (9.5%) was lower than that reported in DESTINY-Breast03 (21%)^35^. CNS as a site of symptomatic progression was very uncommon in the non-BMs cohort of DESTINY-Breast12.

Overall, the safety profile of T-DXd was consistent with previous reports^22,23^, with no new safety signals identified. Discontinuation rates due to AEs were low (15.2% and 9.5% for patients with and without BMs, respectively). Regarding the rates of decreased left ventricular ejection fraction (11.8% and 10.8% for patients with and without BMs, respectively), most cases were grade 1 or grade 2, with only two grade 3 or higher events reported in the BMs cohort.

Lack of an adjudication committee in DESTINY-Breast12 limits direct comparison of ILD/pneumonitis rates with those from previous clinical studies that included an adjudication committee within the protocol. However, rates of ILD/pneumonitis events observed in both cohorts (16% (grade 5: 2%) and 13% (grade 5: 1%) of patients with and without baseline BMs, respectively) were consistent with T-DXd data for patients with HER2^+^ mBC in the DESTINY-Breast01 (16%; grade 5: 3%), DESTINY-Breast02 (10%; grade 5: <1%) and DESTINY-Breast03 (15%; grade 5: 0) studies^35–37^. Most cases of ILD/pneumonitis were mild or moderate; however, six deaths in the baseline BMs cohort and three deaths in the no baseline BMs cohort were judged by investigators to be caused by ILD/pneumonitis. Although opportunistic infections were not systematically tested, five cases of opportunistic infection were reported as co-occurring with ILD/pneumonitis (one grade 4 event and four grade 5 events) in the baseline BMs cohort. Clinical and radiologic features of drug-induced ILD/pneumonitis can resemble infectious etiology^38^; in patients with co-occurring opportunistic infection and ILD/pneumonitis, differentiating the underlying cause of pulmonary toxicity can be challenging, and drug-induced ILD is a diagnosis of exclusion^39^. These results highlight the need to consider PJP prophylaxis in patients taking chronic corticosteroids. Prompt initiation of steroidal treatment in patients with suspected ILD/pneumonitis is required in accordance with current guidelines, and T-DXd should be interrupted as a precaution until the etiology is confirmed^39,40^. Delays in providing this treatment (for example, waiting for results of blood culture tests) should be avoided where possible to minimize worsening of ILD/pneumonitis and associated fatalities in this patient population. Where ILD/pneumonitis is suspected, the possibility of infectious etiology should be explored subsequent to immediate treatment to inform future treatment decisions.

Ongoing studies are further defining the potential CNS efficacy of T-DXd in settings beyond HER2^+^ mBC, including HER2-low breast cancer in the phase 2 TUXEDO-4 study^41^. After the pan-tumor approval of T-DXd in HER2^+^ solid tumors^42^, exploring CNS efficacy of T-DXd outside of breast cancer may be informative. Other ADCs are being tested in prospective clinical studies, including datopotamab deruxtecan in patients with breast cancer and BMs or LM (TUXEDO-2 and DATO-BASE) and patritumab deruxtecan in patients with breast cancer and BMs, non-small cell lung cancer and BMs or solid tumors and LM (TUXEDO-3)^43–45^.

Limitations of DESTINY-Breast12 include the open-label, single-arm study design and exclusion of patients with LM. Few relevant historical cohorts for comparison were available at the time of study protocol development in 2019. Efficacy conclusions relied on single-arm time-to-event efficacy analyses. The immaturity of the final dataset makes cross-trial comparisons challenging, and no long-term follow-up is planned. For the non-BMs cohort, ORR was the primary endpoint despite including patients with no measurable disease at baseline, and PFS was not investigated. A proportion of patients with stable BMs and those with active BMs who were previously treated and progressing had prior CNS radiotherapy, which may have impaired assessment of target lesions. Patients without baseline BMs did not undergo regular brain imaging; therefore, only incidence of symptomatic CNS metastases could be investigated in that cohort. Patients with Black and Asian ethnicities were underrepresented in the treated population. Patient-reported and neurocognitive outcomes were recorded as part of the study, and these analyses will be reported in future reports.

The results of the DESTINY-Breast12 study indicate the CNS efficacy of T-DXd in a large, prospective patient cohort. Without a direct comparison between T-DXd and the tucatinib, trastuzumab and capecitabine regimen, treatment selection for previously treated patients with HER2^+^ mBC and BMs should be balanced between efficacy and toxicity considerations on an individual basis.

In conclusion, T-DXd showed substantial and durable overall and intracranial clinical activity in patients with HER2^+^ mBC, including a large cohort with stable and active BMs. No new safety signals were identified. ILD/pneumonitis remains an important identified safety risk of T-DXd. These results support the use of T-DXd for previously treated patients with HER2^+^ mBC, including those with stable and active BMs.

## Methods

### Inclusion and ethics

This study was approved by the institutional review board or ethics committee at each investigational site before initiation (Supplementary Information). This study was performed in accordance with International Council for Harmonisation Good Clinical Practice guidelines, the Declaration of Helsinki and local regulations on the conduct of clinical research. An independent data monitoring committee was responsible for monitoring patient safety during the study. Patients provided written informed consent before participating in the study. Patients were eligible for inclusion regardless of sex or gender.

### Study design and treatment

We conducted a prospective, open-label, single-arm, multicenter, international phase 3b/4 study involving patients with pathologically documented HER2^+^ advanced or metastatic breast cancer with or without baseline BMs. HER2^+^ expression was locally confirmed as determined by American Society of Clinical Oncology–College of American Pathologists guidelines^46^.

Patients were eligible if they were aged 18 years or older, had disease progression on one or more prior anti-HER2–based regimens, received no more than two prior therapy regimens in the metastatic setting (had to be tucatinib naive) and had an Eastern Cooperative Oncology Group performance status (ECOG PS) score of 0 or 1. Patients with known or suspected LM were excluded.

Patients were allocated to one of two cohorts: those with baseline BMs (previously treated stable BMs and active (untreated or previously treated and progressing) BMs) and those with no evidence of BMs at baseline. Patients with no measurable disease at baseline were permitted to enroll. Stable BMs were defined as BMs radiographically stable for ≥4 weeks since completion of treatment; active BMs were defined as untreated BMs with lesions ≤2 cm or BMs that had progressed since local CNS therapy, with no clinical indication for immediate retreatment with local therapy. Washout periods before the first day of dosing were ≥7 d and ≥21 d for SRS or gamma knife and WBRT, respectively. Patients who received local therapy for isolated CNS progression in either cohort could continue study treatment until a second progression (brain or body) was observed, upon which study treatment was discontinued. Concomitant use of ≤3 mg of dexamethasone daily or equivalent was permitted in patients with baseline BMs. T-DXd was administered intravenously every 3 weeks (21-d cycle) at a dose of 5.4 mg per kg of body weight until RECIST 1.1-defined^47^ disease progression outside the CNS, unless there was unacceptable toxicity or withdrawal of consent or another criterion for discontinuation was met.

Tumor assessments of the chest, abdomen (including the entire liver and both adrenal glands) and pelvis used images from computed tomography (CT) or magnetic resonance imaging (MRI; with intravenous (IV) contrast) collected at screening/baseline and every 6 weeks for the first 48 weeks and 9 weeks thereafter during study intervention. For patients with baseline BMs, MRI (with and without IV contrast) or contrast-enhanced CT images of the brain were collected for all patients at baseline and every 6 weeks for the first 48 weeks and 9 weeks thereafter during study intervention. Patients with active and measurable BMs had intracranial lesions included as target lesion(s) for RECIST 1.1 CNS assessments.

### Endpoints

The primary endpoint for patients with baseline BMs was PFS (time from first dose to disease progression or death (by any cause in absence of progression)). Secondary endpoints included CNS PFS (time from first dose to CNS progression or death); OS (time from first dose to death by any cause); ORR (proportion of patients with confirmed complete or partial response); PFS2 (time from first dose to second progression or death); time to progression (time from first dose until documented disease progression); CNS ORR (proportion of patients with measurable BMs at baseline with confirmed complete or partial response of brain lesions); DOR (time from first documented confirmed response until documented progression or death by any cause); and safety. The primary endpoint for patients with no baseline BMs was ORR. Secondary endpoints included DOR, OS, time to progression, incidence of new symptomatic CNS metastases (number of new symptomatic CNS metastasis during study intervention period / total number of patients without symptomatic CNS metastasis at baseline) and safety. Response and progression in both cohorts were assessed by ICR per RECIST 1.1. Additional prespecified secondary endpoints not reported in this analysis are site of next progression (CNS versus extracranial versus both), duration of treatment on subsequent lines of therapy, patient-reported outcomes (European Organization for the Research and Treatment of Cancer 30-item core quality of life questionnaire (EORTC QLQ-C30), Neurologic Assessment in Neuro-Oncology (NANO) scale, cognitive tests and St. George’s Respiratory Questionnaire–idiopathic pulmonary fibrosis version (SGRQ-I; patients with ILD/pneumonitis only)) in both cohorts and time to new CNS lesions, CNS DOR and MD Anderson Symptom Inventory (MDASI) Symptom Diary (brain tumor-specific outcomes) in the BMs cohort only.

### Safety

AEs were coded using Medical Dictionary for Regulatory Activities version 26.1 preferred terms and graded according to the National Cancer Institute Common Terminology Criteria for Adverse Events (CTCAE) version 5.0. For potential cases of ILD or pneumonitis, study intervention was interrupted and a full investigation was carried out, based on the investigator’s judgment and sponsor review by medical monitor and study safety physician. Adjudication of reported ILD/pneumonitis cases by a separate committee was not conducted in this study; an ILD advisory committee reviewed the diagnosis and management of ILD/pneumonitis cases (outside the parameters of the study). Pulmonary toxicity management guidelines were described previously^48^.

### Statistical analysis

Data analyses were completed using SAS software version 9.4. This single-arm study was not designed to test any prespecified hypothesis; therefore, no formal sample size calculation was performed. The sample size was chosen based on precision estimates for the primary endpoint in each cohort. Assuming an underlying PFS in the BMs cohort and an underlying ORR in the non-BMs cohort in line with available data at the time of study design, a sample of 250 participants in each cohort ensured that the one-sided width of a two-sided 95% CI for each endpoint would not exceed 6.3%. Efficacy analyses were conducted in the full analysis set (defined as all patients who were enrolled in the study and received at least one treatment dose), and no data were excluded. Safety data are reported for the safety analysis set (identical to the full analysis set). Analyses were performed separately by cohort, and no comparison of results between the two cohorts was planned. Safety analyses were descriptive only. PFS, OS, CNS PFS (all 12-month rates), PFS2 and DOR were analyzed by the Kaplan–Meier method. CIs for median PFS were derived based on the Brookmeyer–Crowley method. ORR was assessed using data obtained from first dose until progression, or the last evaluable assessment in the absence of progression, regardless of whether patients withdrew from therapy. CNS ORR was assessed using data obtained from first dose until brain progression, or the last evaluable assessment in the absence of brain progression, regardless of whether patients withdrew from therapy. For PFS, patients who had not progressed or had died by the time of analysis were censored at the time of the latest date of assessment from their last evaluable RECIST 1.1 assessment. Patients who progressed or died immediately after two or more consecutive missed visits were censored at the time of the latest evaluable RECIST 1.1 assessment before the two missed visits. For CNS PFS, patients who had systemic progression, but no CNS progression, were censored at the time of the progression assessment; the analysis did not account for systemic progression as a competing event. For OS, patients not known to have died at the time of analysis were censored on the last recorded date on which the patient was known to be alive. For ORR and CNS ORR, patients who stopped treatment without a response or progression, received a subsequent therapy and then responded were not included as responders. Prespecified subgroup analyses of the full analysis set were conducted for patients with active and stable BMs in the baseline BMs cohort, and descriptive statistics are provided.

### Reporting summary

Further information on research design is available in the Nature Portfolio Reporting Summary linked to this article.

## Online content

Any methods, additional references, Nature Portfolio reporting summaries, source data, extended data, supplementary information, acknowledgements, peer review information; details of author contributions and competing interests; and statements of data and code availability are available at 10.1038/s41591-024-03261-7.

## Supplementary information

Supplementary Information List of investigators and list of independent ethics committees/institutional review boards consulted.

Reporting Summary

## Figures and Tables

**Extended Data Table 1:**
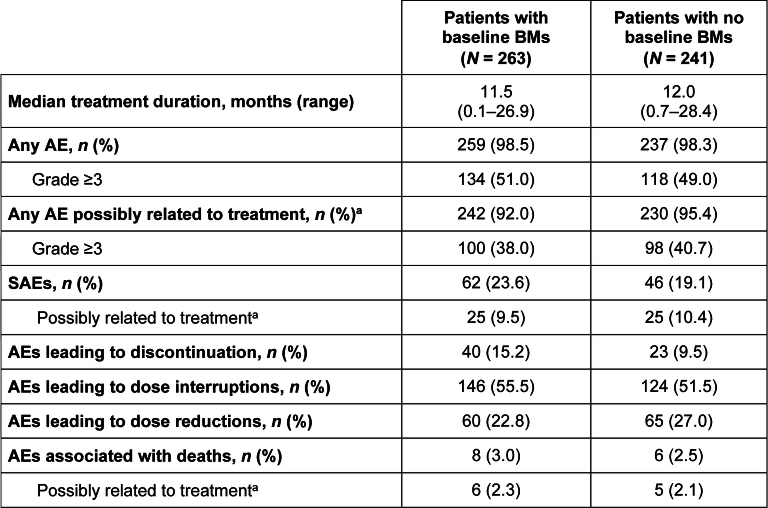
Overall safety summary (safety analysis set)

SAE, serious adverse event.

^a^As assessed by the investigator.

Patients with multiple events in the same category were counted only once in that category. Patients with events in more than one category were counted once in each of those categories.

**Extended Data Table 2:**
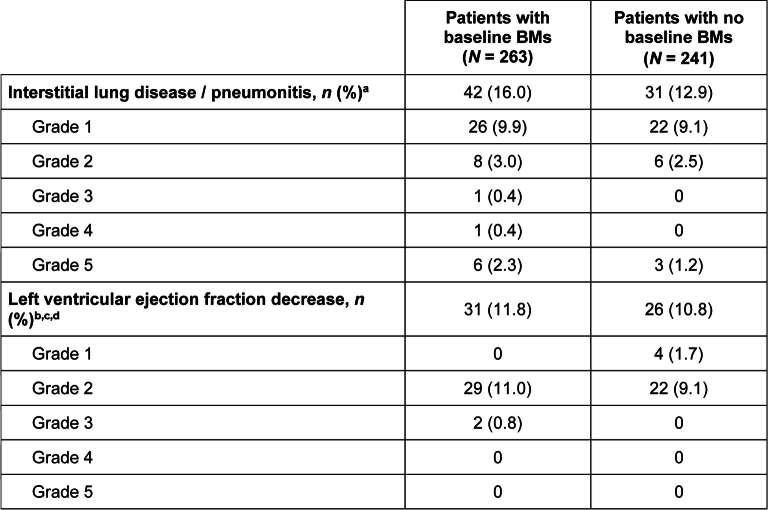
AEs of special interest (safety analysis set)

Each patient is represented with the maximum reported CTCAE 5.0 grade for each grouped term. Patients with events in more than one grouped term are counted once in each of those grouped terms. Includes AEs with an onset date or worsening on or after the date of first dose and up to 47 d after last dose of study treatment or before the start of new anti-cancer therapies, whichever occurred earlier.

^a^Investigator reported, based on preferred terms. Includes 24 (9.1%) pneumonitis (13 (4.9%) grade 1, six (2.3%) grade 2, one (0.4%) grade 3, one (0.4%) grade 4, three (1.1%) grade 5); 17 (6.5%) ILD (12 (4.6%) grade 1, two (0.8%) grade 2, three (1.1%) grade 5); one (0.4%) lung infiltration (grade 1); and one (0.4%) pulmonary fibrosis (grade 1) in patients with baseline BMs. Includes 19 (7.9%) ILD (14 (5.8%) grade 1, four (1.7%) grade 2, one (0.4%) grade 5); 10 (4.1%) pneumonitis (six (2.5%) grade 1, two (0.8%) grade 2, two (0.8%) grade 5); two (0.8%) lung opacity (grade 1); one (0.4%) pulmonary fibrosis (grade 1); and one (0.4%) radiation pneumonitis (grade 1) in patients with no baseline BMs.

^b^Identified based on preferred terms. Includes 29 (11.0%) ejection fraction decreased (27 (10.3%) grade 2, two (0.8%) grade 3); one (0.4%) ischemic contracture of the left ventricle (grade 2); and one (0.4%) left ventricular dysfunction (grade 2) in patients with baseline BMs. Includes 23 (9.5%) ejection fraction decreased (one (0.4%) grade 1, 22 (9.1%) grade 2); two (0.8%) troponin T increased (both grade 2); and one (0.4%) cardiac failure chronic (grade 1) in patients with no baseline BMs.

^c^Two patients discontinued T-DXd treatment due to left ventricular ejection fraction decrease (one in the BMs cohort and one in the non-BMs cohort).

^d^In patients with baseline BMs, the median time to first onset of left ventricular ejection fraction decrease from baseline was 194.0 d (range, 68–506). In patients with no baseline BMs, the median time to first onset of left ventricular ejection fraction decrease was 170.5 d (range, 42–422).

**Extended Data Table 3:**
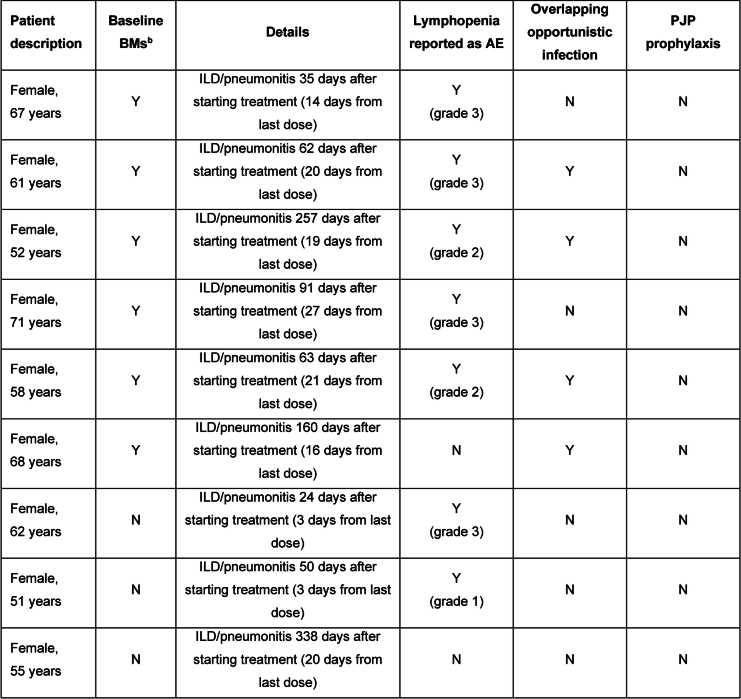
Information on grade 5 investigator-reported cases of ILD/pneumonitis^a^

N, no; NR, not reported; Y, yes.

^a^One patient without baseline BMs had a reported opportunistic infection (grade 5 bronchopulmonary aspergillosis) that was not reported to co-occur with ILD/pneumonitis.

^b^Four patients with baseline BMs had documented use of steroids for BMs, defined as steroid use for ≥1 month or ongoing at data cutoff.

## Data Availability

Data underlying the findings described in this paper may be obtained in accordance with AstraZeneca’s data-sharing policy, described at https://astrazenecagrouptrials.pharmacm.com/ST/Submission/Disclosure. Data for studies directly listed on Vivli can be requested through Vivli at https://vivli.org/. Data for studies not listed on Vivli can be requested through Vivli at https://vivli.org/members/enquiries-about-studies-not-listed-on-the-vivli-platform/. The AstraZeneca Vivli member page is also available outlining further details: https://vivli.org/ourmember/astrazeneca.
