# Publisher Correction: Trastuzumab deruxtecan in HER2-positive advanced breast cancer with or without brain metastases: a phase 3b/4 trial

**DOI:** 10.1038/s41591-024-03349-0

**Published:** 2024-12-09

**Authors:** Nadia Harbeck, Eva Ciruelos, Guy Jerusalem, Volkmar Müller, Naoki Niikura, Giuseppe Viale, Rupert Bartsch, Christian Kurzeder, Michaela J. Higgins, Roisin M. Connolly, Sally Baron-Hay, María Gión, Valentina Guarneri, Giampaolo Bianchini, Hans Wildiers, Santiago Escrivá-de-Romaní, Manoj Prahladan, Helen Bridge, Nataliya Kuptsova-Clarkson, Nana Scotto, Sunil Verma, Nancy U. Lin, the DESTINY-Breast12 study group, Nadia Harbeck, Eva Ciruelos, Volkmar Müller, Naoki Niikura, Christian Kurzeder, Michaela J. Higgins, Roisin M. Connolly, Sally Baron-Hay, Valentina Guarneri, Giampaolo Bianchini, Hans Wildiers, Nancy U. Lin, Belinda Yeo, Nicole McCarthy, Amelia McCartney, Timothy Clay, Nicholas Murray, Andrea Gombos, Joëlle Collignon, Eveline De Cuypere, Katarzyna Jerzak, Stephen Chia, Maja Maraldo, Jeanette Rønlev, Eva Brix, Minna Tanner, Johanna Mattson, Riikka Huovinen, Pauline Wimberger, Mattea Reinisch, Hans Tesch, Michael Untch, Peter Fasching, Tjoung-Won Park-Simon, Joke Tio, Michael Braun, Eva Maria Grischke, Frederik Marmé, Marion van Mackelenbergh, John McCaffrey, Michelino De Laurentiis, Michele Caruso, Rossana Berardi, Laura Biganzoli, Vittoria Fotia, Toshinari Yamashita, Junji Tsurutani, Koichiro Tsugawa, Nobumoto Tomioka, Maaike de Boer, Daniel Houtsma, Martin Pilskog, Olav Engebråten, Anna Sætersdal, Piotr Wysocki, Zbigniew Nowecki, Barbara Radecka, Jacek Jassem, Renata Duchnowska, Joana Simões, Fatima Cardoso, Ana Rita Sousa, María Jesús Vidal Losada, Cristina Saura Manich, Joaquin Gavilá Gregori, Maria Pilar Lopez Marti, Manuel Ruiz Borrego, Javier Cortés Castán, Rafael López López, César Rodríguez Sanchez, Encarnación González Flores, Carmen Hinojo González, Purificación Martínez del Prado, Aglaia Schiza, Fredrika Killander, Leif Klint, Lorenzo Rossi, Stefan Aebi, Khalil Zaman, Peter Hall, Carey Anders

**Affiliations:** 1 Breast Center, Department of Gynecology and Obstetrics, Comprehensive Cancer Center Munich, LMU University Hospital, Munich, Germany; 2 Hospital Universitario 12 de Octubre, Madrid, Spain; 3 CHU Liège and Liège University, Liège, Belgium; 4 University Medical Center Hamburg-Eppendorf, Hamburg, Germany; 5 Tokai University School of Medicine, Kanagawa, Japan; 6 Department of Pathology and Laboratory Medicine, IEO European Institute of Oncology IRCCS, Milan, Italy; 7 Division of Oncology, Department of Medicine 1, Medical University of Vienna, Vienna, Austria; 8 Breast Center, University Hospital Basel, Basel, Switzerland; 9 St. Vincent’s University Hospital, UCD Cancer Trials Cluster, Dublin, Ireland; 10 Cancer Research @UCC, College of Medicine and Health, University College Cork, Cork, Ireland; 11 Cancer Trials Cork, CUH/UCC Cancer Center, Cork University Hospital, Cork, Ireland; 12 Department of Medical Oncology, Royal North Shore Hospital, St Leonards, NSW Australia; 13 IOB-Madrid, Beata María Ana Hospital, Madrid, Spain; 14 Department of Medical Oncology, Ramón y Cajal University Hospital, Madrid, Spain; 15 Department of Surgery, Oncology and Gastroenterology, University of Padova, Padova, Italy; 16 Medical Oncology 2, Istituto Oncologico Veneto IRCCS, Padova, Italy; 17 Department of Medical Oncology, IRCCS Ospedale San Raffaele, Milan, Italy; 18 School of Medicine and Surgery, Vita-Salute San Raffaele University, Milan, Italy; 19 Department of General Medical Oncology, University Hospitals Leuven, KU Leuven, Leuven, Belgium; 20 Vall d’Hebron University Hospital, Vall d’Hebron Institute of Oncology, Barcelona, Spain; 21 Global Medical Affairs, Oncology R&D, AstraZeneca, Cambridge, UK; 22 Oncology Global Medical Affairs / Payer Biometrics, AstraZeneca, Macclesfield, UK; 23 Patient Safety, Oncology R&D, AstraZeneca, Gaithersburg, MD USA; 24 Oncology Global Medical Affairs, AstraZeneca, Baar, Switzerland; 25 Oncology Franchise, AstraZeneca, Gaithersburg, MD USA; 26 Department of Medical Oncology, Dana-Farber Cancer Institute, Boston, MA USA; 27 Austin Health, Melbourne, Australia; 28 ICON Cancer Care, Wesley, Australia; 29 Monash Medical Centre, Clayton, Australia; 30 St John of God, Subiaco Hospital, Subiaco, Australia; 31 GenesisCare, Adelaide, Australia; 32 Institut Jules Bordet, Brussels, Belgium; 33 CHU de Liège, Liège, Belgium; 34 AZ Sint-Jan Brugge-Oostende AV, Bruges, Belgium; 35 Sunnybrook Health Sciences Centre, Toronto, Canada; 36 BC Cancer Agency, Vancouver, Canada; 37 Rigshospitalet, Copenhagen, Denmark; 38 Odense University Hospital, Odense, Denmark; 39 Herlev og Gentofte Hospital, Copenhagen, Denmark; 40 TAYS Sydänkeskus Oy, Tampere, Finland; 41 HUS, Helsinki, Finland; 42 Turku University Hospital, Turku, Finland; 43 Universitätsklinikum Carl Gustav Carus der TU Dresden, Dresden, Germany; 44 Kliniken Essen Mitte, Essen, Germany; 45 Centrum für Hämatologie und Onkologie Bethanien, Frankfurt, Germany; 46 Helios-Kliniken Berlin – Buch, Berlin, Germany; 47 Universitätsklinikum Erlangen, Erlangen, Germany; 48 Medizinische Hochschule Hannover, Hannover Medical School, Hannover, Germany; 49 Universitätsklinikum Münster, Münster, Germany; 50 Rotkreuzklinikum München, Munich, Germany; 51 Universitätsklinikum Tübingen, Tübingen, Germany; 52 Medizinische Fakultät Mannheim der Universität Heidelberg, Heidelberg, Germany; 53 Universitätsklinikum Schleswig-Holstein Campus Kiel, Kiel, Germany; 54 Mater Misericordiae University Hospital, Dublin, Ireland; 55 Istituto Nazionale Tumori Fondazione Pascale IRCCS, Naples, Italy; 56 Humanitas Istituto Clinico Catanese, Catania, Italy; 57 AOU Ospedali Riuniti Umberto I – G.M. Lancisi – G. Salesi, Ancona, Italy; 58 Nuovo Ospedale di Prato, Prato, Italy; 59 A.O. Papa Giovanni XXIII, Bergamo, Italy; 60 Kanagawa Cancer Center, Kanagawa, Japan; 61 Showa University Hospital, Shinagawa, Japan; 62 St. Marianna University Hospital, Kawasaki, Japan; 63 National Hospital Organization Hokkaido Cancer Center, Sapporo, Hokkaido, Japan; 64 Maastricht University Medical Center, Maastricht, Netherlands; 65 HAGA Ziekenhuis locatie Els Borst-Eilersplein, Hague, Netherlands; 66 Helse Bergen HF Haukeland universitetssykehus, Bergen, Norway; 67 Oslo University Hospital, Oslo, Norway; 68 Szpital Uniwersytecki w Krakowie, Krakow, Poland; 69 Narodowy Instytut Onkologii im. Marii Skłodowskiej-Curie, Warsaw, Poland; 70 Opolskie Centrum Onkologii im. prof. T. Koszarowskiego, Opole, Poland; 71 Uniwersyteckie Centrum Kliniczne, Gdansk, Poland; 72 Wojskowy Instytut Medyczny, Warsaw, Poland; 73 Centro Hospitalar Universitario de Porto, Porto, Portugal; 74 Fundação Champalimaud, Lisbon, Portugal; 75 Centro Hospitalar Universitario Lisboa Norte, Lisbon, Portugal; 76 Hospital Clínic Barcelona, Barcelona, Spain; 77 Hospital Universitario Vall d’Hebron, Barcelona, Spain; 78 Fundación Instituto Valenciano de Oncología (IVO), Valencia, Spain; 79 Hospital Universitario de la Princesa, Madrid, Spain; 80 Hospital Universitario Virgen del Rocio, Seville, Spain; 81 Hospital Ruber Internacional, Madrid, Spain; 82 Complejo Hospitalario Universitario de Santiago (CHUS), Santiago, Spain; 83 Hospital Clínico Universitario de Salamanca, Salamanca, Spain; 84 Hospital Universitario Virgen de las Nieves, Granada, Spain; 85 Hospital Universitario Marques de Valdecilla, Santander, Spain; 86 Hospital Civil de Basurto, Bilbao, Spain; 87 Akademiska Sjukhuset Uppsala, Uppsala, Sweden; 88 Skånes Universitetssjukhus, Lund, Sweden; 89 Sahlgrenska Universitetssjukhuset, Göteborg, Sweden; 90 Ente Ospedaliero Cantonale, Bellinzona, Switzerland; 91 Luzerner Kantonsspital (LUKS) - Luzern, Luzern, Switzerland; 92 Centre Hospitalier Universitaire Vaudois, Lausanne, Switzerland; 93 Western General Hospital, Edinburgh, United Kingdom; 94 Duke University Medical Center, Durham, NC USA

**Keywords:** Breast cancer, Breast cancer

Correction to: *Nature Medicine* 10.1038/s41591-024-03261-7, published online 13 September 2024.

In the version of this article initially published, members of the DESTINY-Breast12 study group were not included by the journal in the full text. In Table 1, under the “Prior CNS therapies *n* (%)” section, seven rows have been added, in addition to the original CNS radiotherapy, WBRT and SRS rows. In Table 1 and in the Results: Patients section, “intracranial therapy” has been amended to “CNS radiotherapy,” while the text “the type of intracranial radiotherapy was not always recorded by investigators, and only WBRT and SRS intracranial radiotherapy were reported” has been removed. All changes appear in the HTML and PDF versions of the article.

