# Supplementary material for: Trastuzumab deruxtecan in HER2-positive advanced breast cancer with or without brain metastases: a phase 3b/4 trial
Source: Nat Med. 2024 Sep 13;30(12):3717–27. doi: 10.1038/s41591-024-03261-7 (PMC11645283; doi:10.1038/s41591-024-03261-7)
Supplement: Supplementary file 1 — List of investigators and list of independent ethics committees/institutional review boards consulted. [file 41591_2024_3261_MOESM1_ESM.pdf]

nature medicine

Supplementary information https://doi.org/10.1038/s41591-024-03261-7

Trastuzumab deruxtecan in HER2-positive advanced breast cancer with or without brain metastases: a phase 3b/4 trial

In the format provided by the authors and unedited

Table of contents

List of investigators 2
List of independent ethics committees/institutional review boards consulted 5

List of investigators

Country, study site	Principal investigator
Australia	
GenesisCare	Sally Baron-Hay
Austin Health	Belinda Yeo
ICON Cancer Care	Nicole McCarthy
Monash Medical Centre	Amelia McCartney
St John of God, Subiaco Hospital	Timothy Clay
GenesisCare	Nicholas Murray
Belgium	
Institut Jules Bordet	Andrea Gombos
UZ Leuven	Hans Wildiers
CHU de Liège	Joëlle Collignon
AZ Sint-Jan Brugge-Oostende AV	Eveline De Cuypere
Canada	
Sunnybrook Health Sciences Centre	Katarzyna Jerzak
BC Cancer Agency	Stephen Chia
Denmark	
Rigshospitalet	Maja Maraldo
Odense University Hospital	Jeanette Rønlev
Herlev og Gentofte Hospital	Eva Brix
Finland	
TAYS Sydänkeskus Oy	Minna Tanner
HUS	Johanna Mattson
Turku University Hospital	Riikka Huovinen
Germany	
Universitätsklinikum Carl Gustav Carus der TU Dresden	Pauline Wimberger
Ludwig-Maximilians-Universität München	Nadia Harbeck
Universitätsklinikum Hamburg-Eppendorf	Volkmar Müller
Kliniken Essen Mitte	Mattea Reinisch
Centrum für Hämatologie und Onkologie Bethanien	Hans Tesch
Helios-Kliniken Berlin – Buch	Michael Untch
Universitätsklinikum Erlangen	Peter Fasching
Medizinische Hochschule Hannover, Hannover Medical School	Tjoung-Won Park-Simon
Universitätsklinikum Münster	Joke Tio
Rotkreuzklinikum München	Michael Braun
Universitätsklinikum Tübingen	Eva Maria Grischke
Medizinische Fakultät Mannheim der Universität Heidelberg	Frederik Marmé
Universitätsklinikum Schleswig-Holstein Campus Kiel	Marion van Mackelenbergh
Ireland	
Mater Misericordiae University Hospital	John McCaffrey
Cork University Hospital	Roisin Connolly
St. Vincent’s University Hospital	Michaela Higgins

Italy	
Istituto Nazionale Tumori Fondazione Pascale IRCCS	Michelino De Laurentiis
Istituto Oncologico Veneto IRCCS	Valentina Guarneri
Humanitas Istituto Clinico Catanese	Michele Caruso
AOU Ospedali Riuniti Umberto I – G.M. Lancisi – G. Salesi	Rossana Berardi
Nuovo Ospedale di Prato	Laura Biganzoli
A.O. Papa Giovanni XXIII	Vittoria Fotia
Fondazione San Raffaele Del Monte Tabor	Giampaolo Bianchini
Japan	
Kanagawa Cancer Center	Toshinari Yamashita
Showa University Hospital	Junji Tsurutani
Tokai University Hospital	Naoki Niikura
St. Marianna University Hospital	Koichiro Tsugawa
National Hospital Organization Hokkaido Cancer Center	Nobumoto Tomioka
Netherlands	
Maastricht University Medical Center	Maaike de Boer
HAGA Ziekenhuis locatie Els Borst-Eilersplein	Daniel Houtsma
Norway	
Helse Bergen HF Haukeland universitetssykehus	Martin Pilskog
Oslo University Hospital	Olav Engebråten
Oslo University Hospital	Anna Sætersdal
Poland	
Szpital Uniwersytecki w Krakowie	Piotr Wysocki
Narodowy Instytut Onkologii im. Marii Skłodowskiej-Curie	Zbigniew Nowecki
Opolskie Centrum Onkologii im. prof. T. Koszarowskiego	Barbara Radecka
Uniwersyteckie Centrum Kliniczne	Jacek Jassem
Wojskowy Instytut Medyczny	Renata Duchnowska
Portugal	
Centro Hospitalar Universitario de Porto	Joana Simões
Fundação Champalimaud	Fatima Cardoso
Centro Hospitalar Universitario Lisboa Norte	Ana Rita Sousa
Spain	
Hospital Universitario 12 de Octubre	Eva María Ciruelos Gil
Hospital Clínic Barcelona	María Jesús Vidal Losada
Hospital Universitario Vall d'Hebron	Cristina Saura Manich
Fundación Instituto Valenciano de Oncología (IVO)	Joaquin Gavilá Gregori
Hospital Universitario de la Princesa	Maria Pilar Lopez Marti
Hospital Universitario Virgen del Rocio	Manuel Ruiz Borrego
Hospital Ruber Internacional	Javier Cortés Castán
Complejo Hospitalario Universitario de Santiago (CHUS)	Rafael López López
Hospital Clínico Universitario de Salamanca	César Rodríguez Sanchez
Hospital Universitario Virgen de las Nieves	Encarnación González Flores
Hospital Universitario Marques de Valdecilla	Carmen Hinojo González
Hospital Civil de Basurto	Purificación Martínez del Prado
Sweden	

Akademiska Sjukhuset Uppsala	Aglaia Schiza
Skånes Universitetssjukhus	Fredrika Killander
Sahlgrenska Universitetssjukhuset	Leif Klint
Switzerland	
Ente Ospedaliero Cantonale	Lorenzo Rossi
Universitätsspital Basel	Christian Kurzeder
Luzerner Kantonsspital (LUKS) - Luzern	Stefan Aebi
Centre Hospitalier Universitaire Vaudois	Khalil Zaman
United Kingdom	
Western General Hospital	Peter Hall
United States	
Dana-Farber Mass General Brigham Cancer Care Inc.	Nancy Lin
Duke University Medical Center	Carey Anders

List of independent ethics committees/institutional review boards consulted

Country	Independent Ethics Committee/Institutional Review Board
Australia	Bellberry Limited
	Austin Health Human Research and Ethics Committee
	St John of God Health Care Human Research Ethics Committee
Belgium	Ethische Commissie Onderzoek
Canada	Sunnybrook Health Sciences Centre Research Ethics Board
	University of British Columbia BC Cancer Research Ethics Board
Denmark	De Videnskabsetiske Komiteer For Region Hovedstaden
Finland	Helsingin Ja Uudenmaan Sairaanhoitopiiri
Germany	Ethics Committee at Dresden University of Technology
Ireland	Clinical Research Ethics Committee of the Cork Teaching Hospital
Italy	Comitato Etico IRCCS Pascale
	Comitato Etico per la Sperimentazione Clinica (CESC)
	PO Garibaldi-Centro, ARNAS Garibaldi
	Comitato Etico Regionale (C.E.R.) delle Marche - IRB/IEC
	Comitato Etico Area Vasta Centro
	ASST Papa Giovanni XXIII - Comitato Etico - Bergamo
	Ospedale San Raffaele, IRCCS
Japan	Kanagawa Cancer Center Institutional Review Board
	Showa University Hospital Institutional Review Board
	Tokai University Hospital Institutional Review Board
	St. Marianna University School of Medicine Hospital Institutional Review Board
	National Hospital Organization Hokkaido Cancer Center Institutional Review Board
Netherlands	METC azM/UM - Maastricht UMC+
Norway	REK South-East (Soer-Oest)
Poland	Niezależna Komisja Bioetyczna ds. Badań Naukowych, Gdański Uniwersytet Medyczny
Portugal	CEIC - Comissao de Etica para a Investigaçao Clinica
Spain	Hospital Universitario 12 de Octubre
Sweden	Etikprövningsmyndigheten – Central EC
Switzerland	Ethikkommission Nordwest- und Zentralschweiz
United Kingdom	South Central – Oxford A Research Ethics Committee, Health Research Authority
United States	Dana-Farber Cancer Institute IRB
	Duke University Health System Institutional Review Board
