# Supplementary material for: Trastuzumab deruxtecan in HER2-positive advanced breast cancer with or without brain metastases: a phase 3b/4 trial
Source: Nat Med. 2024 Sep 13;30(12):3717–27. doi: 10.1038/s41591-024-03261-7 (PMC11645283; doi:10.1038/s41591-024-03261-7)
Supplement: Supplementary file 2 — Reporting Summary [file 41591_2024_3261_MOESM2_ESM.pdf]

Corresponding author(s): Nadia Harbeck

Last updated by author(s): Aug 21, 2024

Reporting Summary

Nature Portfolio wishes to improve the reproducibility of the work that we publish. This form provides structure for consistency and transparency in reporting. For further information on Nature Portfolio policies, see our Editorial Policies and the Editorial Policy Checklist.

Statistics

For all statistical analyses, confirm that the following items are present in the figure legend, table legend, main text, or Methods section.

n/a	Confirmed	
☐	☒	The exact sample size (*n*) for each experimental group/condition, given as a discrete number and unit of measurement
☐	☒	A statement on whether measurements were taken from distinct samples or whether the same sample was measured repeatedly
☒	☐	The statistical test(s) used AND whether they are one- or two-sided<br>*Only common tests should be described solely by name; describe more complex techniques in the Methods section.*
☐	☒	A description of all covariates tested
☐	☒	A description of any assumptions or corrections, such as tests of normality and adjustment for multiple comparisons
☐	☒	A full description of the statistical parameters including central tendency (e.g. means) or other basic estimates (e.g. regression coefficient) AND variation (e.g. standard deviation) or associated estimates of uncertainty (e.g. confidence intervals)
☒	☐	For null hypothesis testing, the test statistic (e.g. *F*, *t*, *r*) with confidence intervals, effect sizes, degrees of freedom and *P* value noted<br>*Give P values as exact values whenever suitable.*
☒	☐	For Bayesian analysis, information on the choice of priors and Markov chain Monte Carlo settings
☒	☐	For hierarchical and complex designs, identification of the appropriate level for tests and full reporting of outcomes
☒	☐	Estimates of effect sizes (e.g. Cohen's *d*, Pearson's *r*), indicating how they were calculated

Our web collection on statistics for biologists contains articles on many of the points above.

Software and code

Policy information about availability of computer code

Data collection	Medidata Rave
Data analysis	SAS version 9.4 for statistical analysis

For manuscripts utilizing custom algorithms or software that are central to the research but not yet described in published literature, software must be made available to editors and reviewers. We strongly encourage code deposition in a community repository (e.g. GitHub). See the Nature Portfolio guidelines for submitting code & software for further information.

Data

Policy information about availability of data

All manuscripts must include a data availability statement. This statement should provide the following information, where applicable:

- Accession codes, unique identifiers, or web links for publicly available datasets
- A description of any restrictions on data availability
- For clinical datasets or third party data, please ensure that the statement adheres to our policy

Data underlying the findings described in this manuscript may be obtained in accordance with AstraZeneca's data-sharing policy described at https://astrazenecagrouptrials.pharmacm.com/ST/Submission/Disclosure. Data for studies directly listed on Vivli can be requested through Vivli at www.vivli.org. Data for studies not listed on Vivli could be requested through Vivli at

https://vivli.org/members/enquiries-about-studies-not-listed-on-the-vivli-platform/. AstraZeneca Vivli member page is also available outlining further details: https://vivli.org/ourmember/astrazeneca.

Research involving human participants, their data, or biological material

Policy information about studies with human participants or human data. See also policy information about sex, gender (identity/presentation), and sexual orientation and race, ethnicity and racism.

Reporting on sex and gender	Table 1 reports the sex of the patients. No sex- or gender-based analyses were included in this manuscript because no male patients were included in the study
Reporting on race, ethnicity, or other socially relevant groupings	Table 1 reports the race of the patients
Population characteristics	Population characteristics are reported in Table 1 of the manuscript
Recruitment	Patients were recruited from sites through their circle of care and according to prespecified inclusion and exclusion criteria summarized in the Methods. Reimbursement was available (eg, for transport to sites) to mitigate socioeconomic barriers
Ethics oversight	Institute review boards/ethical committees who approved the protocol are listed in the Supplementary Information

Note that full information on the approval of the study protocol must also be provided in the manuscript.

Field-specific reporting

Please select the one below that is the best fit for your research. If you are not sure, read the appropriate sections before making your selection.

☒ Life sciences ☐ Behavioural & social sciences ☐ Ecological, evolutionary & environmental sciences

For a reference copy of the document with all sections, see nature.com/documents/nr-reporting-summary-flat.pdf

Life sciences study design

All studies must disclose on these points even when the disclosure is negative.

Sample size	This single-arm study was not designed to test any hypothesis; therefore, no formal sample size calculation was performed. The sample size was chosen based on precision estimates for the primary endpoint in each cohort. Assuming an underlying PFS in the BMs cohort and underlying ORR in the non-BMs cohort in line with available data at the time of study design, a sample of 250 participants in each cohort ensured that the 1-sided width of a 2-sided 95% CI for each endpoint would not exceed 6.3%
Data exclusions	Analyses were conducted in the full analysis set, which included all patients treated in the study. No data were excluded
Replication	N/A; this was an analysis of data from a single arm study, therefore there were no replicates
Randomization	Randomization is not relevant to this single-arm study; patients were allocated to cohorts based on absence/presence of brain metastases
Blinding	Investigators were not blinded to the group allocation because this was an open-label, non-randomized, single arm study

Reporting for specific materials, systems and methods

We require information from authors about some types of materials, experimental systems and methods used in many studies. Here, indicate whether each material, system or method listed is relevant to your study. If you are not sure if a list item applies to your research, read the appropriate section before selecting a response.

Materials & experimental systems

n/a	Involved in the study
☐	☒ Antibodies
☒	☐ Eukaryotic cell lines
☒	☐ Palaeontology and archaeology
☒	☐ Animals and other organisms
☐	☒ Clinical data
☒	☐ Dual use research of concern
☒	☐ Plants

Methods

n/a	Involved in the study
☒	☐ ChIP-seq
☒	☐ Flow cytometry
☐	☒ MRI-based neuroimaging

Antibodies

Antibodies used	Antibody-drug conjugate: trastuzumab deruxtecan
Validation	N/A

Clinical data

Policy information about clinical studies

All manuscripts should comply with the ICMJE guidelines for publication of clinical research and a completed CONSORT checklist must be included with all submissions.

Clinical trial registration	NCT04739761
Study protocol	Study protocol was provided with the submission of this manuscript for the purposes of statistical review
Data collection	504 patients were treated across 78 sites between June 2021 and February 2024. Data were collected by site investigators and analyzed and interpreted by the sponsors and authors
Outcomes	The primary endpoint for the baseline BMs cohort was PFS; the primary endpoint for the no baseline BMs cohort was ORR. Additional secondary endpoints were prespecified in the protocol and are reported in the manuscript. Response and progression were assessed by ICR per RECIST 1.1.

Magnetic resonance imaging

Experimental design

Design type	N/A - MRIs determined ICR/investigator-assessed responses (scans were not statistically analyzed individually)
Design specifications	N/A
Behavioral performance measures	N/A

Acquisition

Imaging type(s)	MRI with intravenous contrast
Field strength	As per site - sites were provided with image acquisition guidelines to ensure images were acquired in a standard format
Sequence & imaging parameters	As above
Area of acquisition	Chest, abdomen (including the entire liver and both adrenal glands), pelvis and brain

Diffusion MRI ☐ Used ☒ Not used

Preprocessing

Preprocessing software	N/A
Normalization	N/A
Normalization template	N/A
Noise and artifact removal	N/A

Volume censoring | N/A

Statistical modeling & inference

Model type and settings | N/A

Effect(s) tested | N/A

Specify type of analysis: ☐ Whole brain ☐ ROI-based ☐ Both

Statistic type for inference (See Eklund et al. 2016) | N/A

Correction | N/A

Models & analysis

n/a	Involved in the study
☒	☐ Functional and/or effective connectivity
☒	☐ Graph analysis
☒	☐ Multivariate modeling or predictive analysis
